# Fluorescent Light Energy (FLE) Acts on Mitochondrial Physiology Improving Wound Healing

**DOI:** 10.3390/jcm9020559

**Published:** 2020-02-18

**Authors:** Letizia Ferroni, Michela Zago, Simone Patergnani, Shannon E. Campbell, Lise Hébert, Michael Nielsen, Carlotta Scarpa, Franco Bassetto, Paolo Pinton, Barbara Zavan

**Affiliations:** 1 Maria Cecilia Hospital, GVM Care & Research, 48033 Cotignola (RA), Italy; 2 Department of Medical Sciences, University of Ferrara, 44121 Ferrara, Italy; 3 Klox Technologies Inc., 275 Armand-Frappier Blvd, Laval, QC H7V 4A7 Canada; 4 Department of Morphology, Surgery and Experimental Medicine, Section of Pathology, Oncology and Experimental Biology, Laboratory for Technologies of Advanced Therapies (LTTA), University of Ferrara, 44121 Ferrara, Italy; 5 Klox Technologies Inc., Denmark ApS, Borupvang 5C, 2750 Ballerup, Denmark; 6 Clinic of Plastic and Reconstructive Surgery, Department of Neurosciences, University Hospital of Padova, 35131 Padova, Italy

**Keywords:** fluorescent light energy, inflammation, wound healing, mitochondria, gene expression, mitochondrial dynamics, fluorescence

## Abstract

Fluorescent light energy (FLE) has been used to treat various injured tissues in a non-pharmacological and non-thermal fashion. It was applied to stimulate cell proliferation, accelerate healing in chronic and acute wounds, and reduce pain and inflammation. FLE has been shown to reduce pro-inflammatory cytokines while promoting an environment conducive to healing. A possible mechanism of action of FLE is linked to regulation of mitochondrial homeostasis. This work aims to investigate the effect of FLE on mitochondrial homeostasis in an in vitro model of inflammation. Confocal microscopy and gene expression profiling were performed on cultures of inflamed human dermal fibroblasts treated with either direct light from a multi-LED lamp, or FLE from either an amorphous gel or sheet hydrogel matrix. Assessment using confocal microscopy revealed mitochondrial fragmentation in inflamed cells, likely due to exposure to inflammatory cytokines, however, mitochondrial networks were restored to normal 24-h after treatment with FLE. Moreover, gene expression analysis found that treatment with FLE resulted in upregulation of uncoupling protein 1 (*UCP1*) and carnitine palmitoyltransferase 1B (*CPT1B*) genes, which encode proteins favoring mitochondrial ATP production through oxidative phosphorylation and lipid β-oxidation, respectively. These observations demonstrate a beneficial effect of FLE on mitochondrial homeostasis in inflamed cells.

## 1. Introduction

Wound healing follows a series of complex overlapping processes that leads to closure of the wound and restoration of the epithelial layer, including hemostasis, inflammation, cell proliferation and tissue remodeling [1].

After an injury to skin, the exposed sub-endothelium, collagen and tissue factors activate platelet aggregation to form a clot (hemostasis). Next, neutrophils appear at the site of injury to remove debris and bacteria, promoting an environment for wound healing. Then, macrophages accumulate in the inflammatory phase facilitating phagocytosis of bacteria and damaged tissue. In acute wounds, inflammation is fast and short-lived (typically 3–5 days). The inflammatory cells orchestrate the inflammatory process and facilitate initiation of the reconstructive phase of healing. Lamentably, in chronic non-healing wounds the inflammatory cytokines are in excess, and the resultant pro-inflammatory environment causes degradation of growth factors and extracellular matrix (ECM) proteins [2]. In acute wound, the proliferative phase follows the inflammatory phase, and is characterized by accumulation of fibroblasts and production of connective tissue. Fibroblasts play a critical role in supporting normal wound healing due to their involvement in several key processes, including breakdown of fibrin clot, creation of new ECM and collagen structures, and contraction of the wound [3]. Finally, during the tissue remodeling phase, collagen bundles are reorganized, restoring epidermal barrier function and skin integrity. Overall, the healing process involves numerous cellular and biosynthetic processes, which all require energy in the form of adenosine triphosphate (ATP), as well as amino acids, and other precursor molecules to replace damaged tissue [4,5,6].

Mitochondria are the key organelles responsible for ATP production in human cells, generating energy through oxidative phosphorylation [7]. The mitochondrial respiratory chain consists of a five-subunit complex (I-V) through which electrons are exchanged at increasing reduction potentials, leading to production of ATP [8]. Besides production of ATP for cellular energy, mitochondria also generate reactive oxygen species (ROS) as a by-product of ATP formation [9]. The role of ROS in wound healing is complex. ROS contributes to the oxidative burst that kills bacteria, and acts as a cell signal to increase cell proliferation, which is vital in wound repair [10]. However, ROS are also very potent molecules and can cause damage to DNA, lipids and proteins [11]. Excessive levels of ROS have been associated with chronic wounds, resulting in tissue damage, excessive inflammation, and delayed healing [12,13]. Furthermore, high levels of oxidative stress impact on mitochondrial morphology and positioning within cells, causing mitochondria to cluster around the nucleus as a protective strategy [14,15].

Generally, mitochondria undergo a constant process of fission and fusion where they join and subsequently split back into separate entities. This process is thought to be a housekeeping effect to ensure that mitochondria stay as efficient as possible [16]. The position of mitochondria within cells is dynamic based on localized signals or energy balance. For instance, during cell migration mitochondria will locate close to the migrating cell edges where rapid reorganization of the cytoskeleton filaments requires more ATP for energy [17,18,19].

Photobiomodulation (PBM) is the term used to describe the application of low-level light energy to induce tissue regeneration or to protect tissue that is injured or degenerating [20,21,22]. PBM has been shown to be effective in accelerating healing in chronic and acute wounds [23,24], as well as reducing pain and inflammation in several conditions [25,26,27]. Moreover, PBM reverses toxic effects of neurotoxins, stimulates stem cell proliferation, and displays therapeutic effects in reducing myocardial ischemia reperfusion related to myocardial injury [28,29].

One of the most well-studied mechanisms of PBM is the ability to interact with endogenous chromophores in tissue that absorb photons (light). The most studied endogenous chromophore is complex IV of the mitochondrial respiratory chain, cytochrome c oxidase (CCO). Studies have shown that the activation spectrum of CCO runs from yellow through to red wavelengths (~570–910 nm). Absorption of photons by CCO initiates a biochemical cascade that increases ATP and ROS generation within the electron transport chain [30,31,32,33]. Additionally, evidence suggests that one possible mechanism of action of photons on the wound healing process is regulation of the homeostatic balance between mitochondrial fusion and fission [34]. Mitochondria fusion helps reduce mitochondrial stress by mixing the contents of partially damaged mitochondria. Fission allows the creation of new mitochondria, but it also enables the removal of damaged mitochondria [35].

A form of PBM is fluorescent light energy (FLE) that acts as a vehicle to induce biomodulation. To generate fluorescence, specialized chromophores (light absorbing molecules) are employed to translate light energy into a low-energy emission of fluorescence through a mechanism known as stokes shift [36]. FLE is a unique form of photobiomodulation that has been demonstrated to advance healing of both acute and chronic wounds [37,38]. Studies have demonstrated that acute incisional wounds have reduced inflammation, as well as more physiologic re-epithelization and collagen remodeling resulting in better quality and less visible scars [25,26,27,39]. Additionally, patients with hard-to-heal chronic ulcers experienced accelerated healing and improved quality of life [23]. These studies have documented the safety and efficacy of FLE in a variety of impaired skin conditions [40]. The beneficial impact of FLE on inflammation has been well documented, with improved inflammatory profiles observed in clinical trials for wound healing [37], acne treatment [41], and management of rosacea [42], as well as in canine pyoderma [43] and mechanistic in vitro studies [36]. These studies have demonstrated that FLE has the ability to reduce pro-inflammatory cytokines produced by human dermal fibroblasts (HDFs), as well as increase levels of some anti-inflammatory cytokines, which have a beneficial effect in the treatment of chronic wounds that are stuck in an inflammatory feedback loop [36].

The effect of FLE on mitochondrial homeostasis is still largely unknown. We tested the effect of FLE on an in vitro model of inflammation. Cultures of normal HDFs were stimulated with a cocktail of pro-inflammatory cytokines to create inflamed cells. These inflamed cells were treated with either direct light (photobiomodulation) or FLE. Two forms of photoconverter substrates were tested: an amorphous gel (FLE-Gel) or sheet hydrogel matrix (FLE-Matrix). Variations in the mitochondrial network were analyzed with confocal microscopy. Expression profiles of genes related to mitochondrial dynamic, biogenesis and function were assessed by PCR gene array. Confocal microscopy showed the presence of mitochondrial fragmentation due to exposure to the inflammatory cytokines, but a restoration of the mitochondrial network 24 h post-treatments was observed, in particular in cultures treated with FLE. The gene expression analysis showed that treatment with FLE-Matrix upregulated UCP1 and CPT1B genes, which encode proteins that favor ATP production through oxidative phosphorylation and lipid β-oxidation, respectively. Treatment with FLE-Gel upregulated SLC25A31 gene, linked to cytosolic adenosine diphosphate (ADP) balance. These observations, together with the already known capacity of photobiomodulation to stimulate cytochrome c oxidase, show a beneficial effect of FLE in the treatment of inflamed wounds.

## 2. Experimental Section

### 2.1. Cell Culture Preparation

Normal human dermal fibroblasts (HDFs; PCS-201-012, American Type Culture Collection (ATCC, Manassas, VA, USA) were cultured at 37 °C and 5% CO_2_ in Fibroblast Basal Medium (phenol red-free; ATCC, Manassas, VA, USA) supplemented with Fibroblast Growth Kit-Low serum (ATCC, Manassas, VA, USA). The culture process was performed seeding HDFs in 6-well plastic plates at a density of 10 × 10^4^ cell/well. Healthy cells were incubated in basal medium for the duration of the experiment.

Inflamed cells (including all treatment groups) were incubated for 5–6 h in basal medium prior to inducing an in vitro inflammatory state with an 18-h incubation in an inflammatory cocktail comprising 20 ng/mL each of pro-inflammatory cytokines recombinant human tumor necrosis factor alpha (TNF-α; Miltenyi Biotec S.r.l., Bologna, Italy) and recombinant human Interleukin-1 beta (IL-1β; Miltenyi Biotec S.r.l.).

For all treatment groups (Light, FLE-Gel, and FLE-Matrix), after the 18-h incubation in the inflammatory cocktail, the media was replaced with Phosphate Buffer Saline (PBS; Euroclone S.p.A., Italy) for the illumination procedure (5-min) to minimize scattering or other interference with the light. After the illumination, the PBS was replaced with fresh media containing the TNFα/IL-1β inflammatory cocktail to continue with the inflammatory stimulus. Untreated inflamed HDFs were also placed in PBS for 5 min (to mimic the treatment conditions), and then were incubated in fresh media containing inflammatory cockail for the duration of the experiment.

Mitochondrial morphology was analyzed at 30-min and 24-h post-treatment for all groups, and gene expression profile was investigated at 6-h post-treatment.

### 2.2. Fluorescent Light Energy (FLE) Systems

FLE Systems consist of a multi-LED lamp (KT-L lamp, Klox Technologies Inc., Laval, QC, Canada) and a topical photoconverter substrate in the form of an amorphous gel (FLE-Gel) or sheet hydrogel matrix (FLE-Matrix) (LumiHeal^TM^ Gel and LumiHeal^TM^ Matrix, Klox Technologies Inc., Laval, QC, Canada). The multi-LED lamp delivers non-coherent light between 400–520 nm with a peak at app. 447 nm and a power density between 110–150 mW/cm^2^ at a distance of 5 cm from the *light-emitting diodes* (LEDs). The lamp is equipped with a 5-min timer and a distance indicator. FLE photoconverters contain a chromophore, embedded within the gel or matrix, which can absorb some of the photons from the multi-LED lamp, and emit FLE in the range of approximately 510–700 nm. Thus cells treated with FLE receive a combination of direct light from the multi-LED lamp plus FLE emitted from the Gel or Matrix photoconverter, for delivery of a full spectral range between 400–700 nm. Of note, a dose response for FLE may be observed by assessing FLE-Gel compared with FLE-Matrix, as FLE-Gel generates 0.1–0.2 J/cm^2^ of fluorescence (~510–700 nm) whereas FLE-Matrix generates 0.2–0.7 J/cm^2^.

### 2.3. Fluorescence Light Energy (FLE) Protocols

Three treatment conditions were tested in order to study the impact of FLE on mitochondrial morphology and gene expression. Light-treated cells received a 5-min illumination with the multi-LED lamp placed 5 cm from the bottom of the plate, without the presence of a topical photoconverter. For FLE-treated cells (FLE-Gel or FLE-Matrix) the topical photoconverters were placed under the 6-well plate, not in direct contact with cells, and the multi-LED lamp was placed at 5 cm from the bottom of the plate. Light and FLE are transmitted unchanged through the plastic bottom of the plate, thus contact with the cells is not required to induce their effects. The illumination duration was 5-min for all treatment groups. Each of the following groups were tested:

(a) Healthy: HDFs maintained in basal medium (no inflammatory cocktail or illumination).
(b) Inflamed: HDFs incubated in TNFα/IL-1β inflammatory cocktail.
(c) Light: Inflamed HDFs illuminated for 5-min with only the multi-LED lamp (no FLE).
(d) Gel: Inflamed HDFs illuminated for 5-min with the FLE-Gel system consisting of the multi-LED lamp and topical photoconverter amorphous gel (LumiHeal Gel, Klox Technologies Inc., Laval, QC, Canada).
(e) Matrix: Inflamed HDFs illuminated for 5-min with the FLE-Matrix system consisting of the multi-LED lamp and topical photoconverter sheet hydrogel matrix (LumiHeal Matrix, Klox Technologies Inc., Laval, QC, Canada).
  Healthy HDFs were considered as the control group of the experiment.

### 2.4. Mitochondrial Morphology

Cells were seeded in coverslips 24-mm in diameter and allowed to grow to a confluence of 50–60%. After treatments, cells were fixed with 4% paraformaldehyde solution (Sigma-Aldrich, USA) and washed three-times. Next, cells were permeabilized with a solution of 0.1% triton x-100 (Sigma-Aldrich, USA), for 10 min at room-temperature (RT) on a plate-shaker. After three-washes, unspecific sites were blocked with a solution of 2% bovine serum albumin (Sigma-Aldrich, USA) supplemented of 0.01% triton x-100 for 45 min. at RT with agitation. Cells were next incubated with a primary antibody against TOM20 (mitochondrial marker of the inner membrane) (BD, USA) diluted 1:100 over-night at 4 °C. The next day, cells were washed with three washes of 10 min each with agitation at RT and next incubated with a specific secondary fluorescent antibody Alexa Fluor 488 (Thermo Fisher Scientific, Waltham, USA) diluted 1:1000 in the dark for 45 min at RT with agitation. Cells were acquired in z-stacks of 51 planes at 0.2 µm each at Nikon A1 confocal microscope equipped with a 63X objective. Images obtained were deconvolved to remove blurred signal and 3D reconstructed. The mitochondrial network was then quantified by using the 3D-object counter available in software Fiji (http://fiji.sc/wiki/index.php/Fiji accessed on 29 April 2017) that allow to measure the total object (mitochondria) volume and the number of total objects (mitochondria) per each cell. The mean volume of single mitochondria was calculated by divide the total mitochondria volume with the number of total mitochondria. For each condition, at least 20 cells were analyzed. Data are presented as mean ± SD. Multi comparison statistical analyses were performed by using one-way ANOVA. T test was to perform all pairwise comparisons between group means. Calculated mean ± SD are reported in figure legends.

### 2.5. Total RNA Isolation and PCR Array Profile

As previously described [44], total RNA was extracted by the RNeasy Mini Kit (Qiagen, Hilden Germany) which includes DNase digestion using the RNase-Free DNase Set (Qiagen). For each sample, 500 ng of total RNA were reverse transcribed with RT^2^ First Strand Kit (Qiagen) in SimpliAmp Thermal Cycler (Thermo Fisher Scientific) following the manufacture procedures. Then, the RT^2^ Profiler PCR Array Human Mitochondrial Energy Metabolism (Qiagen) and RT^2^ Profiler PCR Array Human Mitochondria (Qiagen) in StepOne Plus Real-Time PCR System (Thermo Fisher Scientific) were performed. The amplification protocol included the activation at 95 °C for 10 min, followed by 40 cycles of denaturation at 95 °C for 15 s, and elongation at 60 °C for 1 min. The 2ΔΔCT method was used to determine the relative expression of target genes. Cycle threshold (Ct) values of target genes were normalized to the geometric mean Ct values of five housekeeping genes (ACTB: actin, beta; B2M: beta-2-microglobulin; GAPDH: glyceraldehyde-3-phosphate dehydrogenase; HPRT1: hypoxanthine phosphoribosyl transferase 1; RPLP0: ribosomal protein, large, P0). For each target gene, the average of three normalized expression levels were calculated, and *p* values were calculated using Student’s t-test based on 2ΔCT values for each gene in the test group compared to the control group. Statistical significance was set at *p* < 0.05. Results were reported as fold regulation of target genes in test group compared with control group.

## 3. Results

### 3.1. Mitochondrial Morphology Analysis

**Figure 1:**
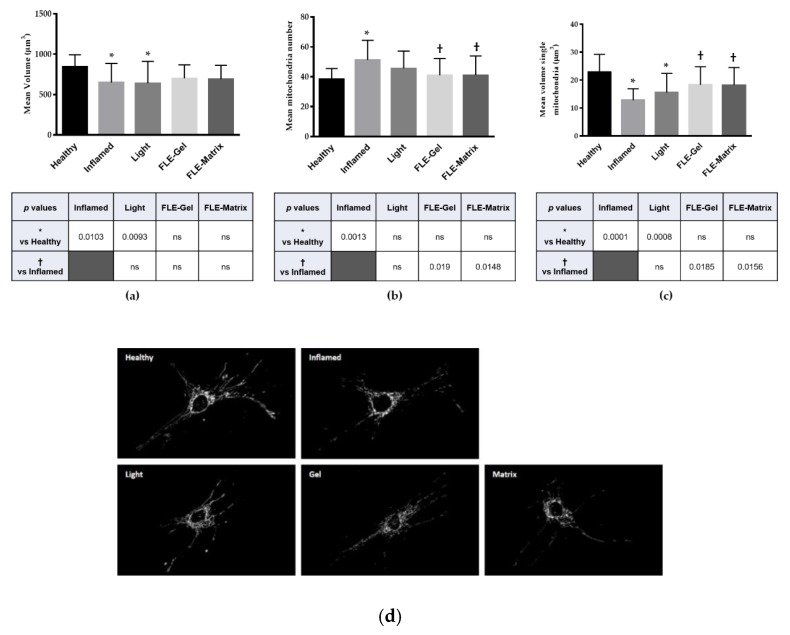
Quantification of mitochondrial network 30-min post-treatment. After deconvolution, images were 3D reconstructed and the mitochondrial network was evaluated by automated estimation of (**a**) volume of the entire mitochondrial network per single cell, (**b**) number of mitochondria per single cell, and (**c**) volume of single mitochondrion. Data are expressed as mean ± SD. Multi comparison statistical analysis were performed by using *one*-*way* analysis of variance (ANOVA). T tests were performed on all pairwise comparisons between group means. * *p* < 0.05 from Healthy human dermal fibroblasts (HDFs), † < 0.05 from Inflamed human dermal fibroblasts (HDFs), ns = not significant. (**d**) Representative images. FLE: fluorescent light energy.

**Figure 2:**
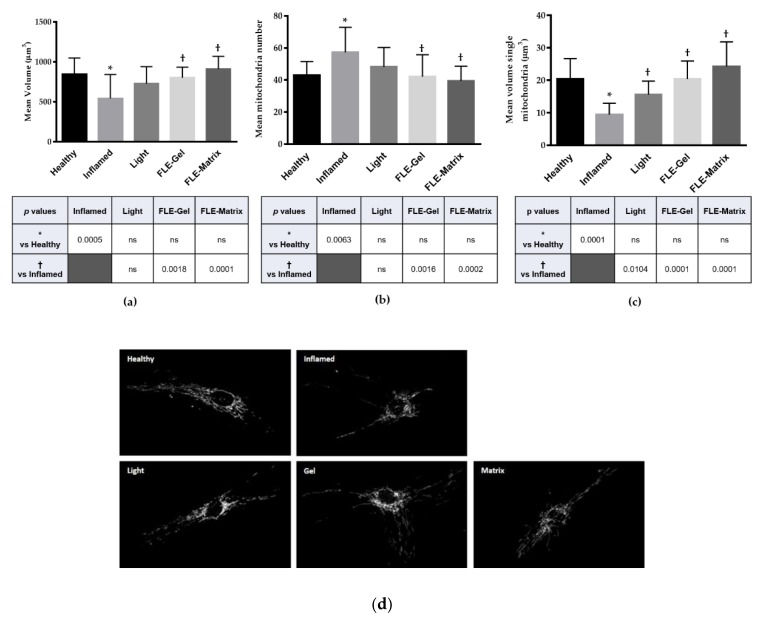
Quantification of mitochondrial network 24-h post-treatment. After deconvolution, images were 3D reconstructed and the mitochondrial network was evaluated by automated estimation of (**a**) volume of the entire mitochondrial network per single cell, (**b**) number of mitochondria per single cell, and (**c**) volume of single mitochondrion. Data are expressed as mean ± SD. Multi comparison statistical analysis were performed by using *one*-*way* analysis of variance (ANOVA). T test was to perform all pairwise comparisons between group means. * *p* < 0.05 from Healthy human dermal fibroblasts (HDFs), † < 0.05 from Inflamed human dermal fibroblasts (HDFs), ns = not significant. (**d**) Representative images. FLE: fluorescent light energy.

Analysis of the mitochondrial morphology was performed in all HDF conditions: (a) healthy HDFs in normal media (Healthy), (b) inflamed HDFs in TNFα/IL-1β inflammatory cocktail (Inflamed), (c) inflamed HDFs treated with light alone (Light), (d) inflamed HDFs treated with FLE-Gel (Gel), and (e) inflamed HDFs treated with FLE-Matrix (Matrix). Importantly, incubation of HDFs with the TNFα/IL-1β inflammatory cocktail led to fragmentation of the mitochondrial network (Figure 1). A global reduction of mitochondrial volume per cell was observed in Inflamed HDFs (Figure 1a) accompanied by an increase in the number of mitochondria per cell, and a reduction of individual mitochondrion volume (Figure 1b,c, respectively). Predictably, greater exacerbation of the mitochondria was observed in Inflamed HDFs at 24 h compare to 30 min, due to the continued exposure to the inflammatory cocktail (Figure 2).

Interestingly, when inflamed cells were exposed to either direct light (Light) or FLE (Gel or Matrix) the mitochondrial network showed preliminary signs of improvement at 30-min post-treatment, with some statistical differences already observed in FLE-treated cells. This improvement occurred despite the continued exposure to the TNFα/IL-1β inflammatory cocktail. Mitochondrial number per cell in Inflamed HDFs was 51 ± 13, compared with 41 ± 11 in FLE-Gel HDFs (*p* = 0.019) and 41 ± 12 in FLE Matrix HDFs (*p* = 0.0148). Similarly, individual mitochondrion volume was 12.8 ± 4.13 µm^3^ in Inflamed HDFs, compared with 18.3 ± 6.5 µm^3^ (*p* = 0.0185) and 18.6 ± 6.3 µm^3^ (*p* = 0.0156) in FLE-Gel and FLE-Matrix HDFs, respectively. Taken together, this data indicates that mitochondria in FLE-treated cells have shifted their mitochondrial network into a more fused state, with larger mitochondria that are fewer in number. While the difference in the volume of the entire mitochondrial network per cell did not reach significance between Inflamed and FLE-treated cells at the 30 min time point, it is interesting to note that while both Inflamed and Light-treated HDFs are different from Healthy HDFs (648 ± 237 µm^3^ and 639 ± 273 µm^3^ vs. 840 ± 153 µm^3^, *p* = 0.0103 and 0.0093 respectively), neither of the FLE-treated groups were significantly different from Healthy HDFs, suggesting they are already starting to recover from the inflamed state.

In order to verify this possibility, the healthiness of the mitochondrial network was analyzed for a longer period of time. At 24-h post-treatment Inflamed HDFs treated with FLE had completely recovered to healthy-HDF mitochondrial morphology, while cells treated with Light were only partially recovered (Figure 2). All observed aspects of the mitochondrial networks were significantly improved in FLE-treated cells compared with Inflamed HDFs. Volume of the entire mitochondrial network per cell was 802 ± 133 µm^3^ in FLE-Gel HDFs, and 910 ± 159 µm^3^ in FLE-Matrix, compared with 523 ± 301 µm^3^ in Inflamed HDFs (*p* = 0.0018 and 0.0001, respectively). Values for the FLE-treated cells were not significantly different from Healthy HDFs (845 ± 205 µm^3^). Additionally, the count of mitochondria number per cell for Inflamed HDFs was 57 ± 16 compared with 48 ± 14 and 39 ± 9 for FLE-Gel and FLE-Matrix (*p* = 0.0016 and *p* = 0.0001, respectively), and individual mitochondrion volume was 9.5 ± 3.5 µm^3^ in Inflamed HDFs compared with 20 ± 5.6 µm^3^ and 24 ± 7.5 µm^3^ for FLE-Gel and FLE-Matrix (*p* = 0.0016 and *p* = 0.0001, respectively). By comparison, Light-treatment had induced some recovery, and was no longer significantly different from healthy HDFs in any parameter. However, it was only significantly improved compared with Inflamed cells in the individual mitochondrion volume (10 ± 3 vs. 16 ± 4 µm^3^ for Inflamed vs. Light respectively, *p* = 0.0104).

**Figure 3:**
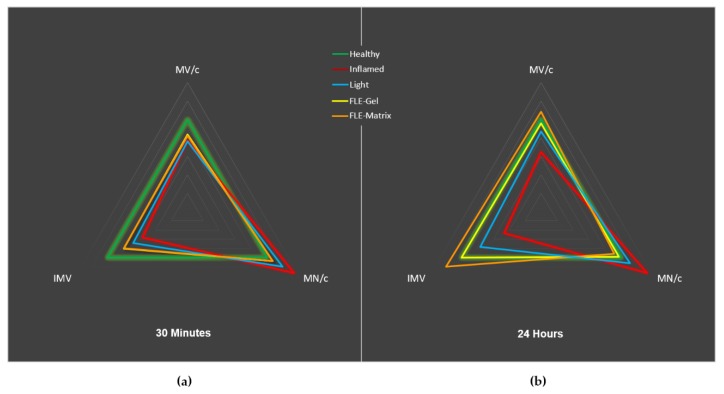
Radial graphs depicting relative changes in mitochondrial networks at (**a**) 30-min and (**b**) 24-h post-treatment. All parameters are indexed to Healthy-HDFs (green). Inflamed-HDFs are depicted in red, Light-treated HDFs in blue, FLE-Gel in yellow, and FLE-Matrix in orange. At time point 30-min the FLE-Gel (yellow) and FLE-Matrix (Orange) overlap, and at time point 24-h the Healthy (green) and FLE-Gel (yellow) overlap. *MV/c = Mitochondria Volume per cell*; *MN/c = Mitochondria Number per cell*; *IMV = Individual Mitochondrion Volume.*

In Figure 3, radial graphs provide a visual representation of the ability of FLE to fully rescue the mitochondrial dynamics of inflamed HDFs. All values are indexed to the healthy values for volume of the mitochondrial network per cell (top, Mitochondria Volume per cell, MV/c), number of mitochondria per cell (bottom right, Mitochondria Number per cell, MN/c) and individual mitochondrion volume (bottom left, Individual Mitochondrion Volume, IMV). The green line represents the Healthy condition, and the red line indicates the Inflamed status. At both the 30-min (Figure 2a) and 24-h (Figure 2b) post-treatment time points, it is apparent that Light-treated cells (blue lines) are shifting away from the Inflamed state towards the Healthy condition. A noticeably greater recovery is visible in FLE-treated cells (FLE-Gel in yellow and FLE-Matrix in orange) at 30-min post-treatment, where both have already progressed beyond the Light cells toward the Healthy condition. Impressively, by 24-h post-treatment FLE-treated cells are completely recovered, even overlapping with the Healthy condition.

### 3.2. PCR Array Gene Expression Profile

In order to investigate the biological impact of the treatments on mitochondrial dynamic and function, mitochondrial gene expression was probed with two different real-time PCR array, measured 6-h post-treatment. 

Table 1 presents data on the expression of 84 key genes associated with mitochondrial respiration, including genes encoding components of the electron transport chain and oxidative phosphorylation complexes. Oxidation of Nicotinamide adenine dinucleotide (NADH) and flavin adenine dinucleotide (FADH2), key metabolites in glycolysis and the citric acid cycle, occurs within a series of four protein complexes embedded in the inner mitochondrial membrane: NADH-coenzyme Q reductase, succinate-coenzyme Q reductase, coenzyme Q-cytochrome c reductase, and CCO. The free energy generated from these processes drives oxidative phosphorylation and ATP synthesis via a fifth protein complex (ATP synthase) [45].

Of note, compared with Healthy HDFs, Inflamed HDFs had upregulated *ATP4A* and *ATP6V0A2* genes and downregulated *COX6C* gene. These genes encode enzymes belonging to ATP synthase and CCO, respectively, which are fundamental for energy production and oxidative phosphorylation. The remaining 81 investigated genes were expressed at similar levels in both conditions. Compared with Inflamed cells, no treatment altered the expression of these genes.

Compared with Inflamed HDFs, cells treated with Light overexpressed two genes belonging to CCO: *COX4I2* and *COX8C.* These genes remained unchanged in both cell group treated with FLE.

Genes belonging to complex I (NADH-Coenzyme Q Reductase), complex II (Succinate-Coenzyme Q Reductase), and complex III (Coenzyme Q-Cytochrome c Reductase) maintained the same expression profile in all the conditions investigated.

Table 2 reports the expression profiles of 84 genes related to biogenesis and function of mitochondria, including regulators and mediators of mitochondrial molecular transport of metabolites required for the electron transport chain and oxidative phosphorylation.

Compared to Healthy cells, Inflamed HDFs showed substantial upregulation of superoxide dismutase 2 (*SOD2*) and neurofilament (*NEFL*) genes. *SOD2* is an antioxidant enzyme that protects cells from oxidative damage, whereas *NEFL* influences the dynamics of mitochondria. Although to a lesser extent, an upregulation of BCL2-antagonist/killer 1 (*BAK1*) and solute carrier family 25, member 25 (*SLC25A25*) and a downregulation of BCL2 binding component 3 (*BBC3*)*,* Cyclin-dependent kinase inhibitor 2A (*CDKN2A*)*,* IMP1 inner mitochondrial membrane peptidase-like *(IMMP1L)*, stratifin *(SFN),* solute carrier family 25 (mitochondrial carrier; citrate transporter), member 1 *(SLC25A1)*, and tumor protein p53 (*PT53*) were also observed. The *BAK1* gene encodes a pro-apoptotic protein. *SLC25A25* and *SLC25A1* genes encode proteins belonging to the family of calcium-binding mitochondrial carriers in the inner membranes of the mitochondria. Their functions are to transport proteins, metabolites, nucleotides and cofactors through the mitochondrial membrane and thereby connect and/or regulate cytoplasm and matrix functions [46]. The *SFN* gene encodes the stratifin protein, a cell cycle checkpoint protein that binds translation and initiation factors and functions as a regulator of mitotic translation. It also regulates signal transduction pathways and cellular trafficking [47]. Cyclin-dependent kinase inhibitor 2A (CDKN2A) is an inhibitor of CDK4 kinase and arrests the cell cycle in G1 phase [48]. The *BBC3* gene encodes the pro-apoptotic protein PUMA that is regulated by the protein tumor suppressor p53 [49]. The *PT53* gene encodes p53 protein that exhibits diverse and global functions, including cell cycle arrest, senescence, and apoptosis. Through these pathways, p53 facilitates the repair and survival of damaged cells or eliminates severely injured cells from the replicative pool to protect the organism. One of the most dramatic responses to p53 activation is the induction of apoptosis [50]. No treatment altered any of the above-mentioned genes compared with Inflamed HDFs.

Of interest, an upregulation of genes involved in the production of ATP and reduction of ROS was observed. Compared to Inflamed cells, Light and FLE-Matrix treated HDFs showed an increase in *UCP1* gene expression. This gene encodes uncoupling protein 1, a member of the family of mitochondrial anion carrier proteins. The uncoupling proteins (UCPs) separate oxidative phosphorylation from ATP synthesis with energy dissipated as heat, also referred to as mitochondrial proton leak. UCPs facilitate the transfer of anions from the inner to the outer mitochondrial membrane and the return transfer of protons from the outer to the inner mitochondrial membrane reducing the mitochondrial membrane potential. A minor decline in the mitochondrial membrane potential leads to a significant decrease in harmful levels of ROS production. UCPs proteins decrease mitochondrial membrane potential to a level still allowing both production of required amounts of ATP, and of lower ROS levels that would be relatively harmless to the cells [51]. Light treated cells also had a reduction in the expression of *SLC25A27*, alias *UCP4*. Moreover, compared to Inflamed HDFs, FLE-Matrix treated cells had an upregulation of *CPT1B* gene. Carnitine palmitoyltransferase-1 (CPT1) is located in the inner aspect of the outer mitochondrial membrane and transports long-chain fatty acids into mitochondria for β-oxidation. The acetyl-coenzyme A (acetyl- CoA) produced by oxidative degradation of fatty acids enters the citric acid cycle for oxidation to carbon dioxide and water by the electron transport chain to yield ATP [52]. Compared to Inflamed cells, the FLE-Gel treated HDFs showed an increase in *SLC25A31* gene expression. The protein encoded by this gene is a member of the ADP/ATP carrier family of proteins that exchange cytosolic ADP for matrix ATP in the mitochondria [53].

## 4. Discussion

In recent years, FLE has been demonstrated to be a safe and effective non-pharmacological intervention for the treatment of acute and chronic wounds [23,37,39] and other skin pathologies [41,42], however, the exact mechanism through which FLE realizes an acceleration in the wound healing process is not yet clear.

The principle behind FLE is the absorption of photons by endogenous chromophores inside the treated tissues, resulting in various biological effects [36]. It is widely known that mitochondria are an initial site of light action in cells, and the central molecule for this is CCO, the terminal enzyme of the mitochondrial respiratory chain. CCO transfers one electron (from each of four cytochrome c molecules), to a single oxygen molecule, producing two molecules of water. At the same time the four protons required are translocated across the mitochondrial membrane, producing a proton gradient that ATP synthase uses to synthesize ATP [54]. CCO has two heme centers and two copper centers. Each of these metal centers can exist in an oxidized or a reduced state, and each are photo-acceptors with different absorption spectra in the red and near-infrared region (up to 950 nm). The absorption of photons by CCO leads to an increase in enzymatic activity, an increase in oxygen consumption, and an increase in ATP production thanks to the photodissociation of inhibitory nitric oxide (NO) [55]. Since NO is non-covalently bound to the heme and copper centers and competitively blocks oxygen at a ratio of 1:10, a relatively low energy photon can move the NO, allowing a lot of respiration to take place [56].

This theory describes the structural changes that CCO undergoes after exposure to photons. Conversely, the present work aims to investigate the mitochondrial morphology and the expression profile of genes related to the respiratory chain, and the mitochondrial dynamics and functions in inflamed cells after the exposure to photons as either direct light (PBM) or FLE. For this purpose, in vitro cultures of HDFs were stimulated overnight with a cocktail of pro-inflammatory cytokines (TNFα/IL-1β) to induce an inflamed state. Then, Inflamed HDFs were treated with one of three different photonic treatments: Light (a non-coherent light between 400 and 520 nm); FLE-Gel of FLE-Matrix (a mix of non-coherent light and FLE with a broad spectrum between 400 and 700 nm). All Inflamed and treated cells were maintained in the inflammatory cocktail environment post-treatment for the duration of the experiment.

Thirty minutes post-treatments, confocal microscopy analysis showed mitochondrial fragmentation in Inflamed HDFs and Light treated cells. Likewise, Motori and colleagues have previously shown that pro-inflammatory stimuli produced localized changes in mitochondrial dynamics, favoring fission over fusion [57]. Indeed, the mitochondrial architecture of a cell results from movement, tethering, fusion and fission events. Owing to frequent fission and fusion, different shapes of mitochondria can be found within a cell, including small vesicles, short rods, and long reticular networks. Strong evidence has demonstrated that mitochondrial dynamics are important for cell viability, senescence, mitochondria health, bioenergetic function, quality control, and intracellular signaling. Shortening of mitochondria is a result of increased fission activity or decreased fusion activity, and is typical for states of reduced bioenergetic efficiency (increased respiratory leak) [58].

Fragmentation of mitochondria may also indicate activation of the autophagic removal of mitochondria by a process known as mitophagy. This process is described as a pro-survival mechanism employed as an early response to cell stress, since it removes damaged mitochondria. Contrarily, if unfavorable condition persists, mitophagy results in cell death. Changes in mitochondrial morphology also occur in the early step of the cell death mechanisms apoptosis and necrosis [59,60]. The progressive loss of mitochondrial network during exposure to inflammatory mediators affirms that chronic inflammation affects mitochondrial morphology and bioenergetic functions. Of note, even as early as 30-min post-treatment, FLE-treated cells were starting to progress toward a healthier state of mitochondria, with significant differences observed between Inflamed HDFs and FLE-treated HDFs in the number of mitochondria per cell and the individual mitochondrion volume.

A full recovery of mitochondrial network occurred by 24-h post-treatment with FLE, manifested by mitochondrial networks comparable to those of Healthy HDFs. A trend towards recovery was also observed with Light-treated cells (PBM), however, while there were no significant differences from Healthy HDFs for mitochondria morphology, only the individual mitochondrion volume was different between Inflamed and Light-treated HDFs. This improved biological effect of FLE compared with PBM has been previously observed [36,40]. While not clearly understood, a possible explanation is biological sensitivity to changes in the light properties that arise during generation of FLE, including polarity, micro-pulsations, or coherency. Of note, opsin proteins are known to be sensitive to light polarity, thus it is feasible that other endogenous chromophores, including CCO, may be as well [61]. This enhanced impact of FLE compared with Light treatment is clearly visible in the radial graphs (Figure 3), with FLE-treated HDFs demonstrating both a quicker and more potent recovery.

Interestingly, we may see a dose-response starting to occur between FLE-Gel and FLE-Matrix. At 30-min, both groups have almost identical mitochondrial dynamics, however, by 24 h it appears as if the FLE-Matrix may be shifting the mitochondria network balance further towards fusion, even beyond that of the Healthy HDFs. However, there is no statistical significance to this finding, so further studies would be needed to better understand any dose effects associated with FLE and its biological implication. It is known that processes associated with increased energy are characterized by mitochondrial elongation and by respiration coupled to ATP synthesis [58]. We can hypothesize that the absorption of low energy photons by CCO has led to a restoration of the mitochondrial network due to an increase in enzymatic activity and respiration, and therefore an increase in ATP production.

Gene expression analysis did not report great alterations in expression of genes encoding enzymes of respiratory chain. In Light-treated cells, compared to Inflamed HDFs, upregulation of *COX4I2, COX8C* and *UCP1* genes was observed. Little is reported about the expression of these genes in the literature, and nothing about their expression during the inflammatory process. However, Suárez and workers linked the upregulation of *COX4I2* gene to the increase in *UCP1* expression in an obese animal model [62]. They demonstrated that the overexpression of the thermogenic factor UCP1 improves metabolic phenotype concurrent with mitochondrial biogenesis trough the *COX4I2* gene overexpression. In Light-treated cells, the improvement in mitochondrial biogenesis due to the upregulation of CCO-coding genes could be the basis of the mitochondrial morphology recorded 24 h post-treatment. In fact, through an increase in the number of mitochondria, a restoration of the mitochondrial volume equal to healthy cells has been observed. Conversely, in Inflamed cells, where *COX6C* gene downregulation was recorded, drastic mitochondrial fragmentation and a decrease in whole mitochondrial volume were found.

It was demonstrated that TNF-α and IL-1β decrease ATP production by reducing the activity of complex I, as well as by reducing mitochondrial membrane potential and inducing mitochondrial DNA damage [63]. The damage and mutations of mitochondrial DNA lead to the synthesis of functionally impaired respiratory chain subunits, promoting increased ROS production. In Inflamed cells, the increased production of ROS was confirmed by the upregulation of *SOD2*, the main mitochondrial antioxidant enzyme. It has been reported that when the inflammatory signal through the NF-κB pathway is strong and extended, the *SOD2* expression is upregulated to maintain ROS homeostasis. However, if the ROS level is not restrained, cell damage and death occur [64]. In Light-treated cells, the levels of ROS are controlled by *UCP1* upregulation via a slight decline in mitochondrial membrane potential, however allowing the production of ATP [44]. Since UCP1 was recently found deregulated during inflammation [65], and essential to preserve mitochondrial structural integrity and function [66,67], we can speculate that Light treatment may improve the mitochondrial function and recover mitochondrial network by the upregulation of this protein.

Instead, treatment with FLE-Matrix resulted in upregulation of *UCP1* as well as *CTP1B*, which is involved in mitochondrial β-oxidation of lipids long-chain fatty acids. Both genes are linked to an increase in ATP production because UCP separates oxidative phosphorylation from ATP synthesis allowing the production of ATP, also in the presence of ROS. Instead CTP1 favors β-oxidation of lipids ensuring the production of ATP [51,52]. It has already been shown that an increase in ATP synthesis is connected with the mitochondrial elongation under different cell conditions [58]. Compared to Light treatment, FLE-Matrix increased the volume of whole mitochondrial network through an increase in the volume of single mitochondria, favoring mitochondrial elongation. Having found the highest mitochondrial network recovery in this condition, we can assume that the gain in ATP production by mitochondrial β-oxidation may help in restoration of mitochondrial dynamics.

Conversely, an increase in *UCP1* and *CTP1B* expression was not found in FLE-Gel treated cells, but an increase in *SLC25A31* gene expression was observed. This gene encodes a nucleotide transporter which imports ADP into the mitochondrial matrix, where it can be converted to ATP by ATP synthase, and then exports the newly synthesized ATP to the cytosol [68]. Therefore, this carrier protein plays an important role in spending the ATP on metabolic processes necessary for cell survival. Since both FLE-Gel and FLE-Matrix recover mitochondrial network, it can be hypothesized that upregulation of this mitochondrial carrier causes an increase in ATP production due to the activation of ATP synthase.

ATP is the cellular energy-carrying molecule essential for multiple cellular functions. Reduced energy levels threaten cellular homeostasis and integrity. Impaired energy metabolism may trigger pro-apoptotic signaling, oxidative damage, cytotoxicity and impede mitochondrial DNA repair [69]. All the evaluated treatments led to an improvement in intracellular ATP levels, thus energizing the cells. However, it seems that FLE treatment rescued inflamed mitochondrial networks, returning them to a healthy condition despite the presence of oxidative stress caused by continued exposure to TNFα/IL-1β inflammatory cytokines. This return of mitochondrial homeostasis by FLE could be a key mechanism supporting accelerated healing and tissue regeneration in wounds and skin pathologies treated with FLE.

## 5. Conclusions

In conclusion, this work investigated the effect of FLE on mitochondrial homeostasis in an in vitro model of inflammation. FLE treatment leads to restoration of the mitochondrial network by 24-h post-treatment, as well as upregulation of *UCP1* and *CPT1B* genes, which encode proteins favoring the production of ATP through oxidative phosphorylation and lipid β-oxidation, respectively. These observations, coupled with the previously established capacity of PBM to stimulate CCO, show a beneficial effect of FLE in the treatment of inflamed wounds. However, these findings derive from an in vitro model and should be validated by an in vivo investigation.

## Figures and Tables

**Table 1:** Human Mitochondrial Energy Metabolism PCR array.

Gene	Inflamed vs. Healthy	*p* Value	Light vs. Inflamed	*p* Value	FLE-Gel vs. Inflamed	*p* Value	FLE-Matrix vs. Inflamed	*p* Value
*ATP12A*	1.08	0.3024	1.31	0.5219	−1.11	0.1645	1.02	0.1648
*ATP4A*	**2.23**	0.7832	1.03	0.6392	−1.03	0.2884	−1.03	0.2967
*ATP4B*	1.45	0.3082	1.79	0.7798	−1.21	0.2096	1.52	0.2148
*ATP5A1*	−1.08	0.9484	−1.14	0.9730	1.06	0.7168	1.03	0.8362
*ATP5B*	−1.05	0.9663	−1.04	0.9969	1.00	0.9906	1.04	0.5569
*ATP5C1*	−1.05	0.9517	−1.12	0.9898	−1.08	0.8672	−1.04	0.9454
*ATP5F1*	−1.23	0.1785	1.01	0.2102	1.05	0.1455	1.07	0.1458
*ATP5G1*	−1.02	0.8216	1.01	0.9565	−1.05	0.1904	1.02	0.2007
*ATP5G2*	−1.15	0.8984	1.28	0.9757	1.28	0.2901	1.08	0.2901
*ATP5G3*	−1.04	0.2888	−1.02	0.8484	−1.09	0.1986	−1.04	0.1983
*ATP5H*	−1.13	0.4352	1.05	0.7275	1.14	0.2591	1.08	0.2589
*ATP5I*	−1.08	0.9752	1.05	0.8689	−1.05	0.8167	0.99	0.9525
*ATP5J*	1.02	0.9968	−1.02	0.9737	−1.04	0.9612	0.99	0.9791
*ATP5J2*	−1.23	0.9810	−1.07	0.9603	−1.01	0.2836	1.22	0.2834
*ATP5L*	1.68	0.5228	−1.36	0.8836	−1.53	0.2096	−1.27	0.2134
*ATP5O*	−1.24	0.8745	1.05	0.7809	−1.03	0.8725	−1.04	0.5821
*ATP6V0A2*	**2.59**	0.9270	1.03	0.3655	−1.79	0.2746	−1.09	0.1516
*ATP6V0D2*	1.91	0.6030	1.62	0.9800	1.36	0.2356	1.21	0.4197
*ATP6V1C2*	1.11	0.1784	1.12	0.6929	−1.18	0.2758	1.20	0.2789
*ATP6V1E2*	1.02	0.6367	1.01	0.8071	1.17	0.1381	1.04	0.1352
*ATP6V1G3*	−1.70	0.4560	1.12	0.8299	−1.11	0.3228	−1.05	0.3231
*BCS1L*	−1.46	0.5451	−1.07	0.8080	1.16	0.4274	1.24	0.4942
*COX4I1*	1.08	0.6312	−1.02	0.6719	1.03	0.7022	1.06	0.7194
*COX4I2*	−1.85	0.9443	**3.24**	0.8211	1.40	0.1855	1.90	0.1854
*COX5A*	−1.15	0.9779	−1.02	0.3636	−1.05	0.1026	1.10	0.0556
*COX5B*	−1.08	0.8219	1.06	0.6803	1.01	0.6248	1.01	0.6164
*COX6A1*	1.05	0.9912	−1.02	0.6452	−1.05	0.9001	−1.06	0.8699
*COX6A2*	1.29	0.7882	1.55	0.4056	−1.03	0.5157	−1.02	0.3269
*COX6B1*	1.09	0.8830	1.06	0.3851	−1.12	0.3845	−1.06	0.1568
*COX6B2*	1.54	0.6464	1.36	0.9643	−1.06	0.2774	1.21	0.2775
*COX6C*	**−2.23**	0.2374	1.12	0.4507	−1.11	0.2241	1.18	0.2246
*COX7A2*	1.31	0.8657	1.00	0.7080	1.00	0.2980	1.02	0.3576
*COX7A2L*	1.14	0.9597	1.18	0.3701	−1.02	0.2149	1.02	0.2810
*COX7B*	−1.23	0.1399	1.03	0.9086	−1.11	0.8089	−1.07	0.8180
*COX8A*	−1.02	0.6213	1.04	0.7314	−1.01	0.3849	1.03	0.4075
*COX8C*	1.11	0.3632	**3.60**	0.3340	−1.20	0.2921	1.67	0.2925
*CYC1*	1.03	0.9564	1.02	0.9543	−1.01	0.8904	1.00	0.8846
*LHPP*	−1.12	0.7322	1.14	0.6447	1.10	0.9146	1.02	0.9953
*NDUFA1*	1.07	0.6609	−1.06	0.7084	1.06	0.2131	−1.16	0.2167
*NDUFA10*	−1.06	0.9250	0.99	0.7389	1.01	0.6267	0.99	0.7675
*NDUFA11*	−1.03	0.9466	−1.02	0.9339	1.04	0.8978	1.09	0.9636
*NDUFA2*	1.07	0.9914	1.02	0.3434	1.11	0.1150	1.01	0.0749
*NDUFA3*	−1.29	0.8155	1.05	0.9753	−1.04	0.8924	−1.03	0.8893
*NDUFA4*	1.09	0.5712	1.03	0.7243	−1.04	0.0812	−1.04	0.0819
*NDUFA5*	−1.97	0.5816	1.28	0.9752	−1.24	0.2903	1.15	0.3374
*NDUFA6*	1.03	0.1884	−1.07	0.5686	1.18	0.1108	1.12	0.1032
*NDUFA7*	−1.03	0.9399	−1.03	0.9026	1.04	0.3395	1.00	0.2997
*NDUFA8*	−1.23	0.8994	−1.08	0.7818	1.23	0.8069	0.99	0.4317
*NDUFAB1*	1.08	0.5897	1.03	0.9813	−1.03	0.7660	−1.04	0.7574
*NDUFB10*	−1.03	0.2064	−1.03	0.6846	1.00	0.7707	−1.02	0.7615
*NDUFB2*	−1.15	0.9577	1.05	0.2898	1.05	0.1720	−1.05	0.3253
*NDUFB3*	−1.14	0.8127	1.03	0.8891	1.07	0.3763	−1.03	0.3061
*NDUFB4*	1.24	0.3245	−1.06	0.7046	1.09	0.0957	1.04	0.0861
*NDUFB5*	−1.07	0.5122	1.04	0.1030	1.05	0.0993	1.03	0.0712
*NDUFB6*	−1.08	0.6834	1.09	0.3958	−1.07	0.4169	−1.21	0.1946
*NDUFB7*	−1.25	0.8837	1.03	0.9139	1.07	0.9146	1.11	0.9221
*NDUFB8*	−1.29	0.9782	−1.08	0.4260	1.04	0.0860	1.04	0.0867
*NDUFB9*	−1.07	0.8154	1.04	0.6321	−1.03	0.3974	1.00	0.3031
*NDUFC1*	−1.13	0.6376	1.06	0.4091	1.10	0.1097	−1.03	0.1077
*NDUFC2*	1.03	0.6633	1.03	0.5582	1.26	0.4491	1.21	0.6712
*NDUFS1*	−1.05	0.8937	1.03	0.8077	1.06	0.2821	1.06	0.2782
*NDUFS2*	−1.05	0.6046	1.08	0.8433	1.08	0.5569	1.05	0.5270
*NDUFS3*	1.14	0.7629	−1.09	0.3173	1.05	0.1314	1.05	0.0777
*NDUFS4*	−1.11	0.3228	1.05	0.6511	−1.01	0.6848	−1.04	0.5687
*NDUFS5*	−1.13	0.9234	1.05	0.8181	1.01	0.3283	−1.03	0.4957
*NDUFS6*	−1.10	0.7346	1.09	0.9146	1.05	0.6572	1.11	0.6882
*NDUFS7*	−1.29	0.7148	−1.32	0.7148	1.05	0.3395	1.02	0.2788
*NDUFS8*	−1.23	0.8670	1.03	0.9886	1.18	0.9448	1.23	0.9275
*NDUFV1*	−1.21	0.8922	0.99	0.6639	1.04	0.1205	1.06	0.1277
*NDUFV2*	−1.21	0.8671	1.08	0.5305	1.06	0.1460	−1.02	0.2872
*NDUFV3*	1.13	0.7272	−1.10	0.3081	1.26	0.1167	1.24	0.1145
*OXA1L*	−1.13	0.5362	1.14	0.5885	1.27	0.1040	1.22	0.1026
*PPA1*	−1.71	0.2951	−1.03	0.5926	1.04	0.8757	−1.02	0.8405
*PPA2*	−1.19	0.5189	1.04	0.4868	−1.02	0.2007	−1.04	0.2018
*SDHA*	−1.42	0.3281	1.09	0.5285	1.05	0.1407	1.02	0.1423
*SDHB*	1.04	0.5917	1.01	0.8479	1.04	0.6252	1.03	0.7692
*SDHC*	−1.19	0.7466	1.22	0.6577	−1.05	0.1798	−1.02	0.1799
*SDHD*	−1.22	0.8702	−1.23	0.9003	1.00	0.5197	−1.07	0.7193
*UQCR11*	1.26	0.4176	−1.16	0.8663	−1.04	0.1969	−1.06	0.1947
*UQCRC1*	−1.03	0.9857	−1.13	0.8575	−1.02	0.9116	−1.04	0.8919
*UQCRC2*	−1.29	0.8090	−1.21	0.1460	1.02	0.1357	1.04	0.1405
*UQCRFS1*	−1.03	0.9645	−1.17	0.8910	−1.03	0.9580	−1.03	0.9512
*UQCRH*	−1.03	0.9254	−1.07	0.9792	−1.04	0.9931	−1.04	0.9962
*UQCRQ*	1.00	0.5753	−1.11	0.7945	1.06	0.8875	1.03	0.8636

ATP12A: ATPase, H+/K+ transporting, nongastric, alpha polypeptide; ATP4A: ATPase, H+/K+ exchanging, alpha polypeptide; ATP4B: ATPase, H+/K+ exchanging, beta polypeptide; ATP5A1: ATP synthase, H+ transporting, mitochondrial F1 complex, alpha subunit 1, cardiac muscle; ATP5B: ATP synthase, H+ transporting, mitochondrial F1 complex, beta polypeptide; ATP5C1: ATP synthase, H+ transporting, mitochondrial F1 complex, gamma polypeptide 1; ATP5F1: ATP synthase, H+ transporting, mitochondrial Fo complex, subunit B1; ATP5G1: ATP synthase, H+ transporting, mitochondrial Fo complex, subunit C1; ATP5G2: ATP synthase, H+ transporting, mitochondrial Fo complex, subunit C2; ATP5G3: ATP synthase, H+ transporting, mitochondrial Fo complex, subunit C3; ATP5H: ATP synthase, H+ transporting, mitochondrial Fo complex, subunit d; ATP5I: ATP synthase, H+ transporting, mitochondrial Fo complex, subunit E; ATP5J: ATP synthase, H+ transporting, mitochondrial Fo complex, subunit F6; ATP5J2: ATP synthase, H+ transporting, mitochondrial Fo complex, subunit F2; ATP5L: ATP synthase, H+ transporting, mitochondrial Fo complex, subunit G; ATP5O: ATP synthase, H+ transporting, mitochondrial F1 complex, O subunit; ATP6V0A2: ATPase, H+ transporting, lysosomal V0 subunit a2; ATP6V0D2: ATPase, H+ transporting, lysosomal 38 kDa, V0 subunit d2; ATP6 V1C2: ATPase, H+ transporting, lysosomal 42 kDa, V1 subunit C2; ATP6V1E2: ATPase, H+ transporting, lysosomal 31 kDa, V1 subunit E2; ATP6V1G3:ATPase, H+ transporting, lysosomal 13 kDa, V1 subunit G3; BCS1L: BCS1-like (S. cerevisiae); COX4I1: Cytochrome c oxidase subunit IV isoform 1; COX4I2: Cytochrome c oxidase subunit IV isoform 2; COX5A: Cytochrome c oxidase subunit Va; COX5B: Cytochrome c oxidase subunit Vb; COX6A1: Cytochrome c oxidase subunit VIa polypeptide 1; COX6A2: Cytochrome c oxidase subunit VIa polypeptide 2; COX6B1: Cytochrome c oxidase subunit Vib polypeptide 1; COX6B2: Cytochrome c oxidase subunit VIb polypeptide 2; COX6C: Cytochrome c oxidase subunit Vic; COX7A2: Cytochrome c oxidase subunit VIIa polypeptide 2; COX7A2L: Cytochrome c oxidase subunit VIIa polypeptide 2 like; COX7B: Cytochrome c oxidase subunit VIIb; COX8A: Cytochrome c oxidase subunit VIIIA; COX8C: Cytochrome c oxidase subunit VIIIC; CYC1: Cytochrome c-1; LHPP: Phospholysine phosphohistidine inorganic pyrophosphate phosphatase; NDUFA1: NADH dehydrogenase 1 alpha subcomplex, 1, 7.5 kDa; NDUFA10: NADH dehydrogenase 1 alpha subcomplex, 10, 42 kDa; NDUFA11: NADH dehydrogenase 1 alpha subcomplex, 11, 14.7 kDa; NDUFA2: NADH dehydrogenase 1 alpha subcomplex, 2, 8 kDa; NDUFA3: NADH dehydrogenase 1 alpha subcomplex, 3, 9 kDa; NDUFA4: NADH dehydrogenase 1 alpha subcomplex, 4, 9 kDa; NDUFA5: NADH dehydrogenase 1 alpha subcomplex, 5, 13 kDa; NDUFA6: NADH dehydrogenase 1 alpha subcomplex, 6, 14 kDa; NDUFA7: NADH dehydrogenase 1 alpha subcomplex, 7, 14.5 kDa; NDUFA8: NADH dehydrogenase 1 alpha subcomplex, 8, 19 kDa; NDUFAB1: NADH dehydrogenase 1, alpha/beta subcomplex, 1, 8 kDa; NDUFB10: NADH dehydrogenase 1 beta subcomplex, 10, 22 kDa; NDUFB2: NADH dehydrogenase 1 beta subcomplex, 2, 8 kDa; NDUFB3: NADH dehydrogenase 1 beta subcomplex, 3, 12 kDa; NDUFB4: NADH dehydrogenase 1 beta subcomplex, 4, 15 kDa; NDUFB5: NADH dehydrogenase 1 beta subcomplex, 5, 16 kDa; NDUFB6: NADH dehydrogenase 1 beta subcomplex, 6, 17 kDa; NDUFB7: NADH dehydrogenase 1 beta subcomplex, 7, 18 kDa; NDUFB8: NADH dehydrogenase 1 beta subcomplex, 8, 19 kDa; NDUFB9: NADH dehydrogenase 1 beta subcomplex, 9, 22 kDa; NDUFC1: NADH dehydrogenase 1, subcomplex unknown, 1, 6 kDa; NDUFC2: NADH dehydrogenase 1, subcomplex unknown, 2, 14.5 kDa; NDUFS1: NADH dehydrogenase Fe-S protein 1, 75 kDa; NDUFS2: NADH dehydrogenase Fe-S protein 2, 49 kDa; NDUFS3: NADH dehydrogenase Fe-S protein 3, 30 kDa; NDUFS4: NADH dehydrogenase Fe-S protein 4, 18 kDa; NDUFS5: NADH dehydrogenase Fe-S protein 5, 15 kDa; NDUFS6: NADH dehydrogenase Fe-S protein 6, 13 kDa; NDUFS7: NADH dehydrogenase Fe-S protein 7, 20 kDa; NDUFS8: NADH dehydrogenase Fe-S protein 8, 23 kDa; NDUFV1: NADH dehydrogenase flavoprotein 1, 51 kDa; NDUFV2: NADH dehydrogenase flavoprotein 2, 24 kDa; NDUFV3: NADH dehydrogenase flavoprotein 3, 10 kDa; OXA1 L: Oxidase (cytochrome c) assembly 1-like; PPA1: Pyrophosphatase 1; PPA2: Pyrophosphatase 2; SDHA: Succinate dehydrogenase complex, subunit A, flavoprotein (Fp); SDHB: Succinate dehydrogenase complex, subunit B, iron sulfur (Ip); SDHC: Succinate dehydrogenase complex, subunit C, integral membrane protein, 15 kDa; SDHD: Succinate dehydrogenase complex, subunit D, integral membrane protein; UQCR11: Ubiquinol-cytochrome c reductase, complex III subunit XI; UQCRC1: Ubiquinol-cytochrome c reductase core protein I; UQCRC2: Ubiquinol-cytochrome c reductase core protein II; UQCRFS1: Ubiquinol-cytochrome c reductase, Rieske iron-sulfur polypeptide 1; UQCRH: Ubiquinol-cytochrome c reductase hinge protein; UQCRQ: Ubiquinol-cytochrome c reductase, complex III subunit VII, 9.5 kDa.

**Table 2:** Human Mitochondria PCR array.

Gene	Inflamed vs. Healthy	*p* Value	Light vs Inflamed	*p* Value	FLE-Gel vs. Inflamed	*p* Value	FLE-Matrix vs. Inflamed	*p* Value
*AIFM2*	1.17	0.9186	1.05	0.9694	1.15	0.8808	1.05	0.3564
*AIP*	−1.12	0.9070	1.16	0.8602	1.21	0.9389	1.13	0.8818
*BAK1*	**2.18**	0.4666	−1.09	0.8772	1.02	0.7111	−1.05	0.1289
*BBC3*	**−2.91**	0.9816	1.31	0.9722	1.08	0.9145	1.04	0.6868
*BCL2*	1.93	0.4330	1.13	0.4945	1.14	0.4947	1.16	0.5045
*BCL2L1*	−1.37	0.9929	1.07	0.4951	−1.07	0.3020	−1.07	0.3250
*BID*	1.51	0.6882	1.01	0.8719	1.06	0.9178	−1.35	0.2987
*BNIP3*	−1.36	0.8372	1.06	0.9668	−1.23	0.9024	−1.16	0.9280
*CDKN2A*	**−2.99**	0.7208	−1.03	0.5949	1.13	0.3788	−1.23	0.3111
*COX10*	−1.03	0.6641	1.01	0.4646	−1.10	0.5937	−1.08	0.3185
*COX18*	−1.36	0.7253	1.32	0.6170	1.30	0.4723	1.09	0.3871
*CPT1B*	−1.33	0.8146	0.99	0.9582	1.82	0.9127	**2.04**	0.4682
*CPT2*	1.11	0.6167	1.11	0.5475	1.04	0.4144	−1.03	0.3732
*DNM1L*	1.04	0.6504	1.02	0.4551	−1.02	0.3857	1.01	0.3536
*FIS1*	−1.29	0.7077	1.12	0.4843	1.15	0.4490	1.07	0.3857
*GRPEL1*	1.40	0.9575	−1.15	0.8785	−1.12	0.3880	−1.01	0.8202
*HSP90AA1*	−1.19	0.8602	1.06	0.5892	1.13	0.1296	1.27	0.4059
*HSPD1*	−1.17	0.8993	−1.01	0.9746	1.00	0.9350	1.07	0.8728
*IMMP1L*	**−2.51**	0.9134	−1.02	0.9908	1.06	0.9928	1.10	0.9780
*IMMP2L*	−1.31	0.5724	1.08	0.9491	1.08	0.9692	1.06	0.4700
*LRPPRC*	−1.20	0.9990	1.19	0.7798	1.07	0.6555	1.10	0.6068
*MFN1*	−1.07	0.7624	1.05	0.9604	1.11	0.8119	1.10	0.5266
*MFN2*	−1.09	0.9807	−1.11	0.7044	−1.15	0.5278	−1.06	0.5023
*MIPEP*	−1.84	0.9939	−1.03	0.8895	1.23	0.9541	1.26	0.4258
*MPV17*	−1.56	0.9050	1.05	0.9991	1.06	0.9772	1.04	0.5992
*MSTO1*	1.04	0.9795	1.10	0.9732	1.26	0.6936	1.17	0.3500
*MTX2*	1.01	0.4066	1.07	0.4383	1.33	0.2515	1.28	0.1868
*NEFL*	**16.93**	0.6088	−1.36	0.9775	−1.65	0.4401	−1.51	0.3560
*OPA1*	−1.20	0.5516	1.15	0.9987	1.10	0.6269	1.15	0.9292
*PMAIP1*	−1.06	0.8934	−1.01	0.9052	−1.24	0.9505	−1.03	0.9594
*RHOT1*	−1.42	0.8552	1.13	0.6221	1.20	0.4302	1.17	0.3495
*RHOT2*	1.07	0.7768	1.03	0.5066	1.00	0.5196	1.00	0.9821
*SFN*	**−2.38**	0.4837	1.66	0.8181	1.90	0.4771	1.75	0.4248
*SH3GLB1*	−1.22	0.9383	1.28	0.9551	1.52	0.8607	1.52	0.3748
*SLC25A1*	**−2.07**	0.8876	1.13	0.8759	1.05	0.7808	1.04	0.7874
*SLC25A10*	1.38	0.6742	−1.62	0.9591	−1.85	0.9908	−1.60	0.9927
*SLC25A12*	−1.26	0.8391	−1.36	0.9808	−1.22	0.9588	−1.12	0.3523
*SLC25A13*	1.51	0.8949	1.11	0.9549	−1.05	0.9313	1.09	0.3061
*SLC25A14*	1.13	0.5778	1.06	0.9728	1.04	0.9826	1.03	0.2791
*SLC25A15*	1.20	0.6720	−1.02	0.5093	1.18	0.4814	1.21	0.3807
*SLC25A16*	−1.12	0.9002	1.13	0.8996	1.03	0.8461	1.16	0.6215
*SLC25A17*	1.26	0.8423	−1.05	0.9918	−1.10	0.8879	−1.06	0.5068
*SLC25A19*	1.44	0.8739	−1.08	0.9144	−1.07	0.8900	−1.06	0.9309
*SLC25A2*	1.14	0.6760	1.17	0.4763	1.15	0.4239	1.48	0.3471
*SLC25A20*	−1.19	0.8596	−1.14	0.7744	1.05	0.8105	1.05	0.5398
*SLC25A21*	−1.42	0.8432	0.99	0.5065	1.30	0.5826	1.43	0.3696
*SLC25A22*	1.21	0.7463	1.07	0.6007	1.11	0.6098	1.17	0.3715
*SLC25A23*	−1.43	0.7167	−1.09	0.4906	−1.52	0.4234	−1.27	0.3587
*SLC25A24*	−1.13	0.8747	1.05	0.8545	1.02	0.9513	1.03	0.3730
*SLC25A25*	**2.30**	0.9570	−1.06	0.9910	−1.11	0.8293	−1.09	0.6173
*SLC25A27*	−1.27	0.3919	**−2.25**	0.9605	−1.10	0.4895	−1.55	0.7273
*SLC25A3*	−1.20	0.3646	1.02	0.7269	1.03	0.4790	1.01	0.1843
*SLC25A30*	−1.36	0.9004	1.12	0.9875	1.08	0.9820	1.10	0.9916
*SLC25A31*	−1.12	0.8581	1.28	0.9795	**2.47**	0.9464	−1.06	0.4897
*SLC25A37*	1.92	0.6504	1.07	0.9261	1.21	0.8353	1.24	0.8423
*SLC25A4*	−1.86	0.6059	1.06	0.8584	−1.03	0.6999	1.02	0.8379
*SLC25A5*	1.00	0.9160	−1.09	0.5923	1.01	0.5904	1.05	0.3863
*SOD1*	−1.14	0.6756	1.09	0.4430	1.08	0.4504	1.14	0.3994
*SOD2*	**17.28**	0.8853	1.28	0.7959	1.19	0.7786	1.13	0.7637
*STARD3*	−1.04	0.3845	1.00	0.8834	−1.02	0.9115	1.01	0.9488
*TAZ*	−1.26	0.8577	1.02	0.3116	1.27	0.2549	1.22	0.9748
*TIMM10*	1.22	0.8033	−1.08	0.5207	−1.09	0.4573	1.03	0.3611
*TIMM10B*	1.14	0.7175	1.06	0.9674	1.01	0.6375	−1.01	0.7582
*TIMM17A*	1.22	0.7552	−1.08	0.7767	1.05	0.7586	1.01	0.7073
*TIMM17B*	−1.33	0.7433	1.29	0.5761	1.15	0.5304	1.23	0.4434
*TIMM22*	−1.06	0.7358	−1.08	0.4982	1.00	0.4019	1.17	0.3696
*TIMM23*	1.07	0.9080	−1.05	0.9481	1.00	0.9475	−1.01	0.7871
*TIMM44*	1.13	0.7086	−1.06	0.5628	−1.14	0.7906	−1.13	0.3392
*TIMM50*	−1.06	0.9866	1.09	0.8853	1.00	0.9968	1.09	0.8634
*TIMM8A*	1.28	0.7994	−1.11	0.1517	−1.05	0.2349	−1.01	0.2730
*TIMM8B*	−1.11	0.9864	−1.06	0.9095	−1.09	0.8707	1.02	0.9847
*TIMM9*	1.37	0.6803	−1.14	0.6204	−1.29	0.4490	−1.33	0.3385
*TOMM20*	1.07	0.9647	1.00	0.9962	−1.05	0.9785	−1.03	0.9860
*TOMM22*	−1.03	0.9395	−1.03	0.9564	−1.05	0.9226	1.00	0.8254
*TOMM34*	1.16	0.9339	1.04	0.9646	0.99	0.9878	1.08	0.9486
*TOMM40*	1.29	0.5140	−1.16	0.6759	−1.09	0.8690	0.99	0.7455
*TOMM40L*	−1.01	0.9861	1.13	0.8819	1.05	0.6619	1.15	0.3629
*TOMM70A*	−1.16	0.9512	1.07	0.9631	1.19	0.8725	1.17	0.3903
*TP53*	**−2.04**	0.7434	1.44	0.8865	1.27	0.5986	−1.05	0.3399
*TSPO*	−1.30	0.8269	1.10	0.5675	1.02	0.4945	1.00	0.3463
*UCP1*	−1.52	0.8095	**3.89**	0.8001	−1.09	0.6418	**2.70**	0.3743
*UCP2*	−1.52	0.9517	1.10	0.9361	1.08	0.8185	1.09	0.3625
*UCP3*	1.59	0.7862	1.64	0.6184	−1.31	0.5903	1.48	0.4868
*UXT*	1.07	0.9794	1.09	0.6893	1.05	0.8536	1.02	0.4300

AIFM2: Apoptosis-inducing factor, mitochondrion-associated, 2; AIP: Aryl hydrocarbon receptor interacting protein; BAK1: BCL2-antagonist/killer 1; BBC3: BCL2 binding component 3; BCL2: B-cell CLL/lymphoma 2; BCL2L1: BCL2-like 1; BID: BH3 interacting domain death agonist; BNIP3: BCL2/adenovirus E1B 19 kDa interacting protein 3; CDKN2A: Cyclin-dependent kinase inhibitor 2A; COX10: COX10 homolog, cytochrome c oxidase assembly protein; COX18: COX18 cytochrome c oxidase assembly homolog; CPT1B: Carnitine palmitoyltransferase 1B; CPT2: Carnitine palmitoyltransferase 2; DNM1L: Dynamin 1-like; FIS1: Fission 1; GRPEL1: GrpE-like 1, mitochondrial; HSP90AA1: Heat shock protein 90 kDa alpha, class A member 1; HSPD1: Heat shock 60 kDa protein 1; IMMP1L: IMP1 inner mitochondrial membrane peptidase-like; IMMP2L: IMP2 inner mitochondrial membrane peptidase-like; LRPPRC: Leucine-rich PPR-motif containing; MFN1: Mitofusin 1; MFN2: Mitofusin 2; MIPEP: Mitochondrial intermediate peptidase; MPV17: MpV17 mitochondrial inner membrane protein; MSTO1: Misato homolog 1; MTX2: Metaxin 2; NEFL: Neurofilament, light polypeptide; OPA1: Optic atrophy 1; PMAIP1: Phorbol-12-myristate-13-acetate-induced protein 1; RHOT1: Ras homolog gene family, member T1; RHOT2: Ras homolog gene family, member T2; SFN: Stratifin; SH3GLB1: SH3-domain GRB2-like endophilin B1; SLC25A1: Solute carrier family 25 (mitochondrial carrier; citrate transporter), member 1; SLC25A10: Solute carrier family 25, member 10; SLC25A12: Solute carrier family 25, member 12; SLC25A13: Solute carrier family 25, member 13; SLC25A14: Solute carrier family 25, member 14; SLC25A15: Solute carrier family 25, member 15; SLC25A16: Solute carrier family 25, member 16; SLC25A17: Solute carrier family 25, member 17; SLC25A19: Solute carrier family 25, member 19; SLC25A2: Solute carrier family 25, member 2; SLC25A20: Solute carrier family 25, member 20; SLC25A21: Solute carrier family 25, member 21; SLC25A22: Solute carrier family 25, member 22; SLC25A23: Solute carrier family 25, member 23; SLC25A24: Solute carrier family 25, member 24; SLC25A25: Solute carrier family 25, member 25; SLC25A27: Solute carrier family 25, member 27; SLC25A3: Solute carrier family 25, member 3; SLC25A30: Solute carrier family 25, member 30; SLC25A31: Solute carrier family 25, member 31; SLC25A37: Solute carrier family 25, member 37; SLC25A4: Solute carrier family 25, member 4; SLC25A5: Solute carrier family 25, member 5; SOD1: Superoxide dismutase 1; SOD2: Superoxide dismutase 2; STARD3: StAR-related lipid transfer (START) domain containing 3; TAZ: Tafazzin; TIMM10: Translocase of inner mitochondrial membrane 10 homolog; TIMM10B: Fracture callus 1 homolog; TIMM17A: Translocase of inner mitochondrial membrane 17 homolog A; TIMM17B: Translocase of inner mitochondrial membrane 17 homolog B; TIMM22: Translocase of inner mitochondrial membrane 22 homolog; TIMM23: Translocase of inner mitochondrial membrane 23 homolog; TIMM44:Translocase of inner mitochondrial membrane 44 homolog; TIMM50: Translocase of inner mitochondrial membrane 50 homolog; TIMM8A: Translocase of inner mitochondrial membrane 8 homolog A; TIMM8B: Translocase of inner mitochondrial membrane 8 homolog B; TIMM9: Translocase of inner mitochondrial membrane 9 homolog; TOMM20: Translocase of outer mitochondrial membrane 20 homolog; TOMM22: Translocase of outer mitochondrial membrane 22 homolog; TOMM34: Translocase of outer mitochondrial membrane 34; TOMM40: Translocase of outer mitochondrial membrane 40 homolog; TOMM40L: Translocase of outer mitochondrial membrane 40 homolog-like; TOMM70A: Translocase of outer mitochondrial membrane 70 homolog A; TP53: Tumor protein p53; TSPO: Translocator protein (18 kDa); UCP1: Uncoupling protein 1; UCP2: Uncoupling protein 2; UCP3: Uncoupling protein 3; UXT: Ubiquitously-expressed transcript.
